# Lamin B Receptor: Interplay between Structure, Function and Localization

**DOI:** 10.3390/cells6030028

**Published:** 2017-08-31

**Authors:** Eleni Nikolakaki, Ilias Mylonis, Thomas Giannakouros

**Affiliations:** 1 Laboratory of Biochemistry, Department of Chemistry, Aristotelian University, 54124 Thessaloniki, Greece; nikol@chem.auth.gr; 2 Laboratory of Biochemistry, Faculty of Medicine, University of Thessaly, Panepistimiou 3 BIOPOLIS, 41500 Larissa, Greece; mylonis@med.uth.gr

**Keywords:** lamin B receptor, LBR, RS domain, SRPK1, Akt, heterochromatin, nuclear envelope

## Abstract

Lamin B receptor (LBR) is an integral protein of the inner nuclear membrane, containing a hydrophilic *N*-terminal end protruding into the nucleoplasm, eight hydrophobic segments that span the membrane and a short, nucleoplasmic *C*-terminal tail. Two seemingly unrelated functions have been attributed to LBR. Its *N*-terminal domain tethers heterochromatin to the nuclear periphery, thus contributing to the shape of interphase nuclear architecture, while its transmembrane domains exhibit sterol reductase activity. Mutations within the transmembrane segments result in defects in cholesterol synthesis and are associated with diseases such as the Pelger–Huët anomaly and Greenberg skeletal dysplasia, whereas no such harmful mutations related to the anchoring properties of LBR have been reported so far. Recent evidence suggests a dynamic regulation of LBR expression levels, structural organization, localization and function, in response to various signals. The molecular mechanisms underlying this dynamic behavior have not yet been fully unraveled. Here, we provide an overview of the current knowledge of the interplay between the structure, function and localization of LBR, and hint at the interconnection of the two distinct functions of LBR.

## 1. Introduction

**Figure 1:**
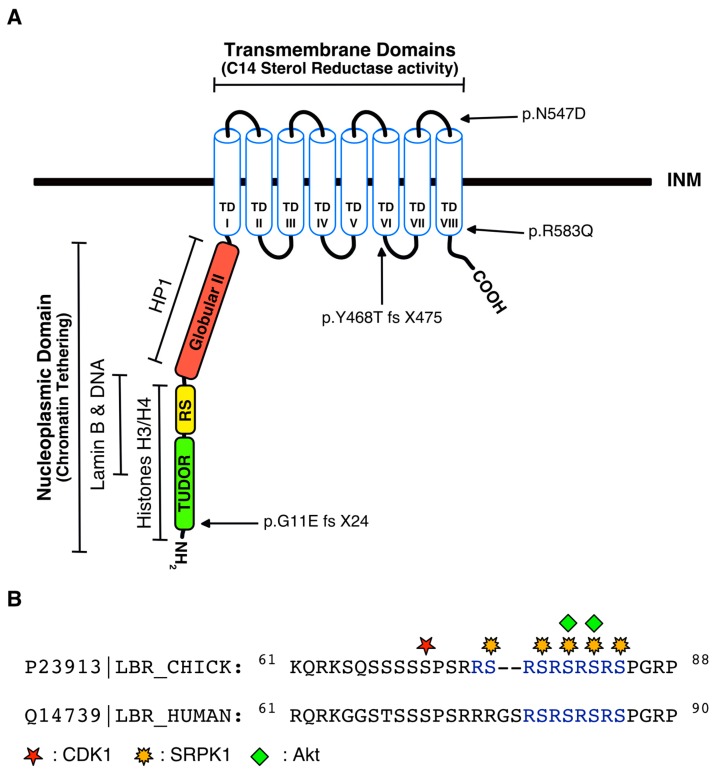
(**A**) Schematic illustration of full-length LBR. Green, yellow and red boxes correspond to tudor (Met 1 to Phe 60), arginine/serine (RS; Arg 61 to Arg 89) and second globular regions (Pro 90 to Gly 211 in human LBR), respectively, that compose the hydrophilic nucleoplasmic domain, while TDI–TDVIII represent the eight transmembrane domains where the sterol reductase activity of LBR resides. The binding sites for histones H3/H4, DNA, lamin B and HP1 are illustrated. Arrows indicate the positions of the missense mutations R583Q and N547D that result in the production of full-length LBR which lacks sterol reductase activity, and the frame shift mutations p.G11E fs X24 and p.Y468T fs X475 that create premature stop codons in codons 24 and 475, respectively; (**B**) Alignment of the chicken and human RS domain sequences. Numbers indicate the number of amino acid residues from the *N*-terminus. Red, yellow and green marks indicate the serine residues in chicken LBR phosphorylated by CDK1, SRPK1 and Akt kinases, respectively.

A defining feature of eukaryotic cells is the presence of a membrane-enclosed nucleus. The nuclear envelope (NE) that surrounds the nucleus is composed of the outer nuclear membrane (ONM), which faces the cytoplasm and is continuous with the rough endoplasmic reticulum (ER), and the inner nuclear membrane (INM), which faces the nucleoplasm and is underlain by a filamentous meshwork, the nuclear lamina. The INM and the nuclear lamina, besides their pivotal contribution in nuclear architecture, also play a key role in chromatin organization and gene regulation by providing a platform for clustering specifically modified chromatin [1,2,3]. Genes in the NE-associated chromatin areas are typically repressed, while artificial tethering experiments suggest that proteins of the INM and the lamina can contribute to this repression [1,2,4]. Over 100 transmembrane proteins are found embedded and/or retained at the INM [5]. Only a few of these proteins have been characterized so far, and these studies bring up some interesting issues [6,7,8,9,10]. The intrinsic INM proteins are often organized in large multimeric complexes, associate both with lamina and chromatin, and also mediate the anchoring of a variety of regulatory proteins at the nuclear periphery. LBR is probably the most well-studied integral protein of the INM (Figure 1A). 

LBR is one of the most important proteins of the INM, mediating the peripheral tethering of heterochromatin at early developmental stages [4] and in cycling cancer cells [11]. On the other hand, on the basis of the sterol C14 reductase activity exhibited by its transmembrane domains, it has been proposed that many human cells in culture depend on LBR to produce cholesterol [12]. In this review, we summarize the progress that has been made in our understanding of the molecular mechanisms underlying the complex crosstalk between structure, function and localization of this multifaceted INM component, whilst also trying to address the question of why evolution “created a chimeric protein”, piecing together a sterol metabolism enzyme and an INM membrane architectural protein.

## 2. LBR Targeting to the Nuclear Envelope

INM proteins are synthesized on the surface of the ER membrane, from which they move laterally by diffusion to the ONM. They then diffuse without restriction between the ONM and the INM through the nuclear pores, but are preferentially retained at the INM due to interactions with nuclear components such as chromatin and/or lamins [13,14,15]. Recent observations from Ungricht et al. [16] and Boni et al. [17] support such a diffusion–retention-based mechanism for targeting LBR to the inner nuclear membrane. While soluble macromolecules pass through the central channel of the nuclear pores with the assistance of importins and nucleoporins carrying phenylalanine–glycine (FG)-rich repeats, LBR and other integral INM proteins pass through peripheral channels [16,17,18]. The nucleoporin53–93 subcomplex facilitates the translocation process by properly forming the peripheral gateways for INM proteins, while energy in the form of ATP is also required [16,17,19].

According to the initial studies from Smith and Blobel [13] and Soullam and Worman [14], two structural features of LBR jointly contribute to its NE localization. The first element resembles a bipartite nuclear localization signal (NLS) and consists of two basic segments (from Arg 63 to Arg 79 and from Arg 93 to Lys 108 in chicken LBR) in the *N*-terminal nucleoplasmic domain. The second targeting signal resides in the first transmembrane domain (residues 241–246 of chicken LBR). Based on these data, a GFP-fusion protein (LBR1TM-GFP) encompassing the *N*-terminal domain and the first transmembrane segment of human LBR (residues 1–238) has been widely used as a reporter to follow the association with the NE [15,16,17,20]. Recent data from Giannios et al. confirmed that the *N*-terminal targeting signal (from Gln 62 to Glu 112 in human LBR) is essential for translocation and retention of LBR into the cell nucleus [21]. However, even if residues within this domain are engaged in interactions with heterochromatin and/or lamin B, these interactions on their own are not sufficient to concentrate LBR at the nuclear periphery, and a transmembrane domain is also required [21]. It does not have to be necessarily the first transmembrane domain, as initially suggested by Smith and Blobel [13] and Soullam and Worman [14], since a GFP-fusion protein containing the whole *N*-terminal nucleoplasmic domain and transmembrane domain VI, instead of transmembrane domain I, exhibited similar properties with LBR1TM-GFP when assayed by fluorescence recovery after photobleaching (FRAP) analysis [21].

Another interesting issue arising from the study of Giannios et al. [21] is that the diffusional mobility of LBR differed significantly along the NE, depending on which subregion of the NE was probed. This regional variation is indicative of a non-uniform distribution of LBR along the INM [21,22]. Furthermore, various amino-terminal and transmembrane deletions affected differently LBR dynamics at the nuclear envelope, either reducing or increasing its diffusional mobility, suggesting that LBR configuration is fine-tuned by a complex network of structural features and interactions between various domains [21].

## 3. Regulation of LBR by Post-Translational Modifications

Phosphorylation of the arginine/serine (RS) region, which consists of four (human LBR) or five (avian LBR) consecutive RS dipeptides and is located at the nucleoplasmic *N*-terminal domain (Figure 1B), plays critical roles in the regulation of LBR structure and localization. Initially, an LBR-associated kinase targeting the RS repeats was purified from turkey erythrocytes and partially characterized [23]. This RS kinase activity was subsequently identified as SRPK1, and was shown to phosphorylate all serine residues within the RS domain of LBR [24,25] (Figure 1B). A highly conserved RSRSPGR peptide sequence, partially overlapping with the RS repeats, serves as a docking motif of SRPK1. This peptide, as suggested by molecular dynamics simulations, may promote unfolding of the adjacent RS repeats prior to their phosphorylation [26]. The serine residues of the *C*-terminal RS dipeptides (especially Ser80 and Ser82 in chicken LBR, see Figure 1B) that conform to the consensus recognized by Akt kinases (RXRXXS/T) are also targeted by Akt1 and Akt2 [27]. Moreover, according to the Scansite Motif Scanner (http://scansite.mit.edu/motifscan_seq.phtml), a motif-profile-scoring algorithm generated by Yaffe and Cantley which takes into consideration not only the phosphorylation motif but also the influence of the flanking residues [28], the serine residues of the RS region are all considered as potential targets of CLK kinases. CLK-mediated phosphorylation is very likely to occur in vivo, since CLK kinases efficiently phosphorylate RS domain-containing proteins [29,30].

LBR molecules have the tendency to self-associate in vitro through their RS domains and form oligomers that are mostly insoluble [22,31,32,33]. A considerable portion of native LBR at the INM also resides at closely spaced but distinct microdomains, which are clearly detectable by both conventional and super-resolution light microscopy [21,22]. The existence of these microdomains that contain predominantly immobile “aggregated” LBR molecules, and areas of the INM that contain mostly mobile, non-oligomerized LBR molecules, probably accounts for the differences in diffusional mobility obtained in fluorescence recovery after photobleaching and fluorescence loss in photobleaching (FRAP/FLIP) assays [21]. The entrapped molecules in the microdomains are better retained in the INM and diffuse with difficulty, whereas the “free” LBR molecules shift continuously throughout the NE–ER. Phosphorylation alters the RS repeats’ conformation, and induces the formation of an Arg-claw-like structure exposing phosphate groups to the periphery [26], very similar to the structure found in the case of the phosphorylated RS domain of SRSF1 (ASF/SF2) [34]. In vitro, this structural alteration results in the dissociation of the oligomers with a concomitant increase in solubility [31,33]. Thus, it is reasonable to speculate that phosphorylation of the RS dipeptides may regulate the oligomerization state of LBR in vivo. In line with this hypothesis, it was recently reported that ELYS, a component of the nuclear pore complex (NPC), regulates the localization of LBR by modulating its phosphorylation state [35]. ELYS depletion by specific siRNAs inhibited the activity of protein phosphatase 1, and accordingly promoted the phosphorylation of serine residues within the RS domain. As a result, LBR was not confined to the nuclear envelope, and became dispersed throughout the ER. On the contrary, the nuclear envelope localization of LBR was maintained upon treatment of cells with SRPIN340, a specific inhibitor of SRPKs [36]. Interestingly, the observed mislocalization was restricted to LBR, as other INM proteins, such as emerin and Lap2, were not obviously affected by ELYS depletion [35].

One intriguing question concerns the proportion of LBR molecules that are phosphorylated, as well as the stoichiometry of the phosphorylation of every single LBR molecule. Given that the vast majority of confocal images from normal and cancer cells in interphase show that LBR localizes to the nuclear rim [see indicatively refs [4,15,21], we estimate that the extent of phosphorylation is limited, and that the majority of LBR molecules are multimerized and organized in microdomains. In line with this suggestion, both SRPK1 and Akt kinases are mainly present in the cytoplasm, and their translocation to the nucleus is tightly controlled by specific upstream signaling events [37,38,39,40]. On the contrary, LBR becomes hyperphosphorylated at the beginning of mitosis. An unknown cell cycle-dependent signal triggers the translocation of SRPK1 to the nucleus at the G2/M boundary, while the kinase also becomes upregulated [37,41]. Furthermore, the most prominent mitotic kinase, cdk1, phosphorylates Ser71 (in both human and chicken LBR) just upstream of the RS domain [42,43] (Figure 1B). These combined phosphorylation events probably result in the complete dissociation of the oligomeric structures, render LBR highly mobile, and promote the disassembly of the NE. 

While phosphorylation has been extensively studied, very little is known about other post-translational modifications of the protein. There is only one report on the O-β-linked *N*-acetyl-glucosaminylation (O-GlcNAcylation) of a serine residue (Ser 96) downstream of the RS domain of rat LBR [44]. The cross-talk of this modification with phosphorylation is unknown, while this serine residue is not conserved in homologous avian or human LBR. Further studies are therefore required to unravel the significance of O-GlcNAcylation, and to see whether LBR molecules from other species can also be O-GlcNAcylated and under what cellular conditions. 

## 4. The Intricate Roles of LBR at the Nuclear Envelope

### 4.1. LBR Tethers Heterochromatin to the INM in Undifferentiated Cells

**Figure 2:**
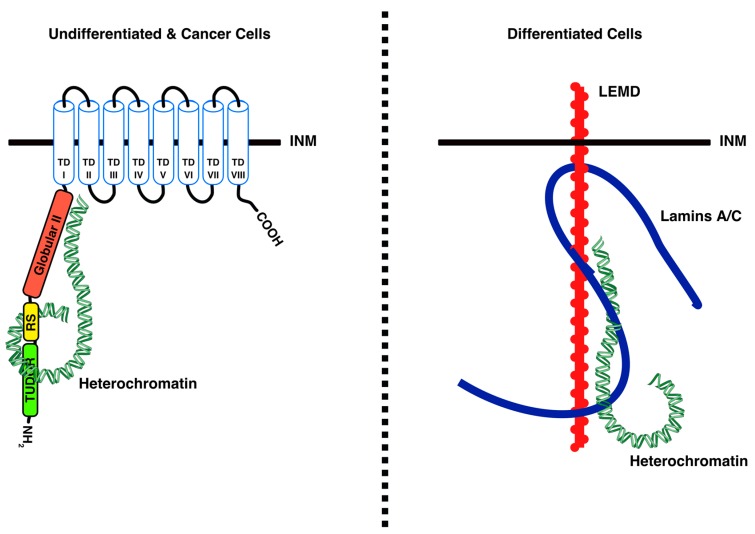
Schematic illustration of heterochromatin tethering to the INM. LBR directly tethers heterochromatin in undifferentiated and cancer cells (left panel), while lamin A/C indirectly tethers heterochromatin in differentiated cells, probably through the LEMD ([LAP2/emerin/MAN1] domain-containing) proteins. From Solovei et al. [4] with permission from Elsevier.

The highly-ordered organization of chromatin constitutes a basic feature of nuclear architecture and plays an important role in the regulation of gene expression [45]. Most eukaryotic cells display a conventional nuclear organization with heterochromatin underlying the INM and the nucleoli, whereas euchromatin is located at the nuclear interior [4,46,47]. Recently, Solovei et al. demonstrated that peripheral tethering of heterochromatin occurs via LBR in early developmental stages and via lamin A/C in differentiated cells [4] (Figure 2). While LBR binds chromatin directly [6,22,25,32,48,49], lamin A/C functions as a chromatin tether indirectly, probably through other chromatin-interacting proteins, such as LEMD ([LAP2/emerin/MAN1] domain-containing) proteins [8,10,50,51]. Importantly, in cells lacking both LBR and lamin A/C, peripheral heterochromatin cannot be maintained and becomes clustered in the nuclear interior [4]. The so-called “inverted nuclear organization”, where heterochromatin condenses in the nuclear center and euchromatin is displaced to the periphery, occurs naturally in rod photoreceptors of nocturnal mammals, in which both LBR and lamin A/C are missing [52]. Ectopic expression of LBR in these cells counteracts the inverted nuclear organization and results in chromatin relocalization to the nuclear periphery [4]. Furthermore, in mouse cells which do not express lamin A/C, such as lymphocytes, knockdown of LBR leads to internalization of peripheral heterochromatin [4].

A similar chromatin relocalization occurs in mouse olfactory sensory neurons (OSNs). In this system, only one out of 2800 olfactory receptor (OR) genes is expressed per olfactory neuron, while the remaining receptor genes, which are located at different chromosomes, aggregate into large nuclear foci, thus ensuring their effective silencing [53]. The heterochromatinization of OR genes is concomitant with a global internalization of peripheral heterochromatin, and is a result of LBR downregulation as the neurons develop [53]. Restoration of LBR expression to OSNs reverses the inverted nuclear organization and peripheral heterochromatin is moved towards the nuclear periphery. As a consequence, OR gene aggregation is disrupted and a large number of ORs are expressed. Furthermore, in an olfactory placode-derived cell line (OP6), which appears to be an intermediate state between a typical non-OSN cell and a fully differentiated OSN, LBR is diffusely distributed inside the nucleus [54]. The dispersed nuclear localization of LBR is associated with relocation of peripheral heterochromatin to the nuclear interior along with LBR [54].

An exception to the model that LBR is expressed in undifferentiated or early differentiated states, while the expression of lamin A/C is linked to differentiation, is seen in the nuclear morphology of blood granulocytes [6]. During the process of granulopoiesis, the levels of lamin A/C are significantly reduced, whereas LBR is strongly upregulated. Concurrently, the nucleus undergoes indentation, and chromosomal regions are displaced from the nuclear interior into the nuclear periphery [55,56,57]. The lobulated nuclear shape facilitates the deformation of neutrophils, the most abundant type of granulocytes, through narrow spaces of tissues, while their motility would be severely hindered by a round-shaped typical nucleus [58]. In LBR knockdown cells, the differentiated granulocyte nuclei become ovoid with redistributed heterochromatin [56]. These knockdown cells provide an in vitro model for genetic disorders such as the Pelger–Huët anomaly in humans [59] and ichthyosis in mice [60,61], both of which are caused by mutations in the *LBR* gene leading to a complete or partial lack of functional LBR. The nuclei from neutrophils of individuals who are carriers of the Pelger–Huët anomaly are hyposegmented [57,59], while homozygous ichthyosis mice exhibit ovoid neutrophil nuclei on blood smears with a characteristic inverted nuclear organization.

LBR also tethers heterochromatin to the INM in cycling cancer cells [11] (Figure 2). Interestingly, LBR was shown to prevent cellular differentiation, whereas lamin A/C promoted it [4,62]. In addition, certain types of cancer cells have reduced levels of lamin A/C as compared with their nonmalignant progenitors [63,64]. Downregulation of LBR by small hairpin RNAs led to the relocation of heterochromatin from the nuclear periphery to the nucleoplasm [11]. Furthermore, similarly to the differentiation state of normal cells, induction of cellular senescence by oncogene activation or by γ-irradiation significantly decreased the levels of LBR, while heterochromatin detached from the INM and condensed in the nuclear interior into irregular structures resembling cords [11,65]. Yet, according to another report, LBR was redistributed in the nucleoplasm and also in the cytoplasm of Hela cells following 5-bromodeoxyuridine-induced senescence [66]. In all three reports, a significant decrease of lamin B expression was observed, implying that the regulation of both LBR and lamin B is interrelated. Importantly, the reduction (or mislocalization) of LBR and lamin B, and the consequent relocation of chromatin to the nuclear interior, are consequences and not causes of senescence. The senescence phenotype was not manifested in cell lines with reduced LBR and lamin B expression, and other factors (such as DNA damage) were required to trigger senescence [11].

Finally, LBR was shown to specifically associate with the Xist long noncoding RNA, and to thus tether the X chromosome to the INM and inactivate it during development in female mammals [67,68]. The recruitment of the X chromosome to the nuclear periphery changes its overall structure, enabling Xist and its associated transcriptional repressors to silence transcription. 

### 4.2. LBR–Chromatin Interactions and Functional Consequences

LBR binds to chromatin via its first globular/tudor (from Met 1 to Phe 60 in human LBR) and RS (from Arg 61 to Arg 89 in human LBR) domains (see Figure 1). Deletion of either the tudor or the RS domain reduced binding to chromatin and increased the diffusional mobility of the respective LBR mutants at the NE [20,21,32,69]. Chromatin pulled-down by LBR was enriched in trimethylated Lys 9 and Lys 29 histone H3 (H3K9me3, H3K29me3), which are considered as typical heterochromatin marks [22]. LBR probably stabilizes/maintains heterochromatin structure by inducing chromatin compaction [32]. Compaction is mediated by the specific interaction of dimethylated Lys 20 histone H4 (H4K20me2) with the tudor domain and the multimerization activity of the RS domain [32]. The requirement of multimerized RS domains to observe compacted chromatin suggests that the RS dipeptides are mainly dephosphorylated, since phosphorylation results in the dissociation of oligomers [31,33,35]. Studies on the disassembly and reassembly of NE during mitosis further confirm the suggestion that phosphorylation inhibits the chromatin binding activity of LBR. At the beginning of NE breakdown, hyperphosphorylated “free” and highly mobile LBR molecules dissociate from chromatin and disperse in the ER [15,25,43]. The high extent of phosphorylation also functions as a switch, preventing premature membrane assembly around chromosomes [43]. In late anaphase, PP1/2A dephosphorylates LBR, which allows oligomerization of the RS domains and subsequent chromatin association [43,70].

Peripheral localization of chromatin principally coincides with transcriptional silencing [71]. In line with these earlier observations, Hirano et al. demonstrated that GAL-4 fused LBR repressed transcription of a reporter plasmid [32]. Transcriptional repression was a consequence of chromatin compaction, but also required recruitment of transcriptional modulators/repressors, such as HP1, MeCP2 and possibly lamin B. HP1 is a strong transcriptional repressor which binds to the second globular domain (from Pro 90 to Gly 211 in human LBR) [72,73], while MeCP2—which interacts with Sin3A, thereby recruiting histone deacetylases—and lamin B bind to both the tudor and RS domains [69,74] (see Figure 1A). In this respect, deletion of either the second globular domain, which does not seem to function in chromatin compaction, or the tudor and RS domains, significantly reduced the transcriptional repression activity of LBR.

Thus, the nucleoplasmic domain of LBR binds to, compacts and silences chromatin by concealing the transcription factor binding sites and recruiting transcriptional modulators. Yet, it is rather unlikely that the nucleoplasmic domain of a single LBR molecule can bind concomitantly to adjacent nucleoplasmic domains [22,31,32,33], DNA [31,48,49], histones H3 and H4 [22,32,67,75], lamin B [23,32,48,67,76] and transcriptional repressors [32,69,71,74]. The RS region, for example, which is only 30 amino acids in length, mediates multimerization of the *N*-terminal domain, but is also implicated in interactions with DNA, histones H3 and H4, lamin B and MeCP2. Even if the respective binding sites are partially overlapping, some of these interactions are most likely mutually exclusive due to steric hindrances. This leads us to support the hypothesis that within microdomains, various LBR molecules are engaged in different interactions, which are dictated by their extent of phosphorylation. Unphosphorylated LBR molecules are mostly engaged in multimerization [31,32,33] and nucleosome association via DNA [31,48] and/or specifically modified histones [32]. On the other hand, partially phosphorylated molecules are less multimerized and can bind lamin B [42,77] and transcriptional repressors. We also need to consider the possibility that less multimerized and/or non-multimerized molecules may also operate as transient docking sites for free histones before they get incorporated into heterochromatin [69,78] and protamine, prior to their deposition on sperm chromatin [79]. In addition, they may interact with ELYS, in the context of reformation of the nuclear pore complex at the end of mitosis [33], and importin β, during the NE assembly [20]. 

### 4.3. LBR C14 Sterol Reductase Activity

LBR can also function as an enzyme catalyzing the reduction of the C14-unsaturated bond of lanosterol, as part of the metabolic pathway leading to cholesterol synthesis [6]. The *C*-terminal transmembrane domains of LBR share extensive sequence homology with another C14 sterol reductase called TM7SF2 (also known as DHCR14 or SR-1) [80]. In addition, ectopic expression of human LBR can complement C14 reductase mutants of *Saccharomyces cerevisiae* [81] and *Neurospora crassa* [82]. TM7SF2, like most of the enzymes involved in the cholesterol biosynthesis pathway, localizes to the ER membranes, and its expression is regulated by the sterol regulatory element-binding protein (SREBP) in response to cellular cholesterol levels; whereas LBR is an inner nuclear membrane protein, and the *LBR* gene lacks an SRE consensus sequence, thus being constitutively expressed [12,83]. Disruption of the gene encoding TM7SF2 in mice does not impair cholesterol synthesis, implying that LBR complements C14 sterol reductase activity in TM7SF2-/-mice [84]. By contrast, in homozygous ichthyosis mice that lack LBR, TM7SF2 seems to be inadequate to compensate for the LBR deficiency [61]. Strikingly, on the basis of structural similarities between an integral membrane sterol reductase from *Methylomicrobium alcaliphilum* and the enzyme isoprenylcysteine carboxyl methyltransferase, Li et al. recently suggested that the sterol reductase domain of LBR may, in addition, recognize and carboxymethylate the farnesylated cysteine of substrates such as prelamin A and/or lamin B [85]. 

Over the last few years, an increasing body of data has led to the notion that the C14 sterol-reductase activity of LBR is distinct from its chromatin-anchoring properties and is critical not only for certain cell functions but also for cell viability [12,86]. Tsai et al. demonstrated that many human cells strictly depend on LBR to produce cholesterol [12]. One critical issue not addressed in the studies supporting that the features of LBR thought to mediate chromatin tethering can be uncoupled from their role in cholesterol biosynthesis [12,86,87] pertains to the dynamic changes in chromatin organization in cells lacking a functional LBR. Tsai et al. examined only NE morphology which was not affected by the lack of LBR [12]. However, Lukášová et al. clearly demonstrated that, while the phenotypic differences between parental HeLa cells and cells in which LBR was knocked down were minor, the reduced expression of LBR resulted in the relocation of heterochromatin from the INM to the nucleoplasm and was associated with its unfolding [11]. Related to this, puzzling differences in cell growth and viability were observed as a result of LBR knockdown in various human cells in culture. According to Lu et al., knockdown of LBR by siRNA caused HeLa cell death in the early G1 phase via apoptosis [20]. Cells could be rescued by expression of exogenous full-length LBR, but not by its *C*-terminal transmembrane domains, where the sterol reductase activity resides. Lukášová et al. showed that shRNA-mediated reduction of LBR expression in U2OS and MCF7 by 78 and 69%, respectively, resulted in significantly slower proliferation rates than the parental cells [11]. Finally, no differences in cell growth were observed by Tsai et al. between parental and LBR-knockout HeLa, HEK293T and human foreskin fibroblasts cells (complete knockdown using the CRISPR/Cas9 system) under normal growth conditions [12]. Only under cholesterol-restrictive growth conditions did LBR knockout cells exhibit reduced proliferation rates, cell rounding and detachment, followed by cell death. Cells could be rescued by expression of either full-length LBR or the *C*-terminal sterol reductase domain [12]. These notable discrepancies may reflect the decisive influence of chromatin structural organization on human cell viability. It is critical to determine whether the non-affected cell lines described by Tsai et al [12] have an inverted architecture with heterochromatin localizing to the nuclear interior, or whether these cells retain a layer of heterochromatin tethered to the nuclear periphery by lamins and other integral INM proteins, thus resembling differentiated cells [4].

### 4.4. Disease-Associated Mutations in the LBR Gene

LBR mutations cause the human Pelger–Huët anomaly [59], the human Greenberg skeletal dysplasia which is lethal [87,88,89] and ichthyosis in mice [61]. Heterozygous LBR mutations lead to nuclear hyposegmentation of neutrophils without causing disease [6,59,87], while homozygous LBR mutations cause various malformations ranging from cardiac defects, brachydactyly and mental retardation (homozygous Pelger–Huët anomaly), severe skin disease (ichthyosis in mice) and prenatal death (Greenberg dysplasia) [6,87]. Mutations resulting in the Pelger–Huët anomaly are identified everywhere in the gene, while mutations causing Greenberg skeletal dysplasia are mainly found in the *C*-terminal region resulting in abnormality of the hydrophobic transmembrane domains [90,91]. Since mutations causing the Pelger–Huët anomaly result in bilobed neutrophil nuclei in heterozygotes and unsegmented, ovoid nuclei in homozygotes, the Pelger–Huët anomaly has been characterized mainly as a laminopathy [6,56,59]. On the contrary, as mutations associated with Greenberg skeletal dysplasia result in a deficiency of sterol reductase activity and in elevated levels of sterol intermediates, Greenberg dysplasia has been often characterized as a disease of cholesterol metabolism [12,87,89,91].

Mutations in the *LBR* gene can be categorized into two types: missense point mutations, such as R583Q and N547D [87] (see Figure 1A), and base pair insertions or deletions causing frame shifts that create premature stop codons, as for example in codons 24 and 475 of human LBR [87] (see Figure 1A) and in codons 175 and 386 of mouse LBR [61]. Missense mutations result in the production of full-length LBR which, however, is practically devoid of sterol reductase activity, since both these amino acid substitutions map to the NADPH binding pocket and significantly reduce its reductive capacity [12,85]. Mutations creating premature stop codons result in truncated forms of LBR, in which several transmembrane helices or even larger parts of the molecule are omitted. Interestingly, it was recently shown that LBR truncation mutants are highly unstable and are rapidly turned over [12].

Truncated forms of LBR—depending on the half-life of the mutated protein and whether mutations are heterozygous or homozygous—lead to significantly reduced amounts of LBR at the INM, that may cause severe malformations ranging from hyposegmented nuclei in neutrophils and the Pelger–Huët anomaly to Greenberg dysplasia [87,91]. Similarly, mutations in the *LBR* gene that result in complete loss of its sterol reductase activity [12] are developmentally lethal in humans. In this respect, homozygosity for mutation N547D was associated with Greenberg dysplasia [87,89,92]. Of particular interest is the missense mutation R583Q. The individual who was heterozygous for this mutation had well-lobulated neutrophils and did not show any evidence for the Pelger–Huët anomaly [87]. On the basis of this data, it was suggested that the structural role of LBR in INM was distinct from its sterol reductase activity [12,87,91].

## 5. Our Current View and Perspectives

The nuclear organization of chromatin regions is highly ordered and has a functional impact on gene regulation [45,93,94,95,96,97]. Heterochromatin primarily resides at the nuclear periphery, whereas euchromatin predominantly occupies internal nuclear regions. Differentiation of embryonic stem cells provides a typical example of the link between chromatin architecture and gene expression. Undifferentiated pluripotent stem cells possess dispersed chromatin within the nuclear interior with limited compaction, while differentiated cells possess large compact chromatin domains associated with the nuclear envelope [98]. Furthermore, targeting genes to the nuclear envelope can strongly affect transcription [71,99,100,101]. Therefore, the association of a gene locus with a particular nuclear neighborhood may be the cause of gene activation or repression, rather than the consequence. In fact, it has recently been suggested that the higher-order folding of chromatin topology may act as a molecular pathway independent code regulating gene expression [102].

**Figure 3:**
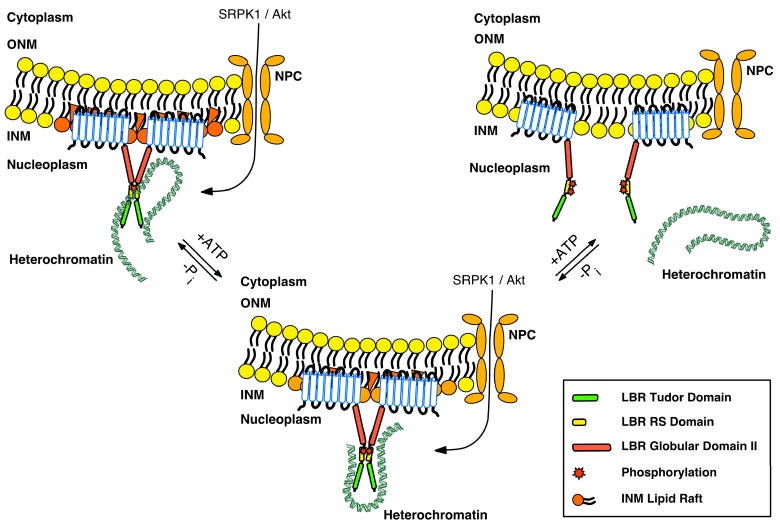
A provisional model for the LBR–heterochromatin interaction. LBR exists in oligomers that reside in distinct microdomains embedded within specific INM lipid rafts. LBR is mainly unphosphorylated and associates with heterochromatin through its tudor (green box) and RS (yellow box) domains. The second globular domain (red box) may also contribute, to some extent, to heterochromatin association via HP1. Activation of SRPK1 and/or Akt kinases, combined with their nuclear translocation due to various stimuli, modifies the oligomerization state of LBR, and accordingly, its binding to various partners of the LBR complex. We hypothesize that at the same time, the composition of LBR-associated lipid rafts also changes, either as a direct effect of phosphorylation or via other signals that regulate the sterol reductase activity and may or may not be related to phosphorylation. These concomitant alterations result in chromatin rearrangement and altered gene expression. Hyperphosphorylation of LBR by SRPK1 and Akt (and cdk1 at the beginning of mitosis) results in the dissociation of oligomers that are no longer embedded in lipid rafts, and the consequent dissociation of heterochromatin. “Free” LBR molecules exhibit an increased diffusional mobility throughout NE–ER.

We think that LBR is a key component of the NE, and coordinates gene expression in undifferentiated cells in response to various stimuli. LBR exists in oligomers that reside in distinct microdomains at the INM. The microenvironment of a given microdomain conforms to the various molecular forms and oligomeric states of LBR, due to phosphorylation, possibly O-GlcNAcylation and/or other as-yet uncharacterized post-translational modifications, and the consequent cross-interaction between the LBR binding partners, combined with the interactions with other NE components. The available information on how cell signaling affects the organization of peripheral heterochromatin is very limited. SRPK1, which is the main kinase targeting RS dipeptides, has been considered as a kinase highly specific for the SR family of splicing factors, and most of the existing literature focuses on splicing [29,30]. SRPK1 has been recently reported to be overexpressed in multiple cancers and exhibit pleiotropic effects that have been attributed to disturbed alternative splicing, which is a common feature of human tumors [103,104]. In addition, Akt1 and Akt2 kinases have received much attention in the study of cancer because they are key mediators of the PI3K-signaling pathway, which is involved in the regulation of cell cycle, proliferation, apoptosis, protein synthesis and glucose metabolism [39,105]. Interestingly, many cancer cells exhibit increased nuclear size, irregular nuclear contours and disturbed chromatin distribution, making nuclear morphology one of the oldest and most commonly used cancer markers [63,106,107]. Thus, overexpression of SRPK1 and/or activation of Akt1/2, combined with their potential nuclear translocation due to cancer-related signaling events [30,39,40,105], may account for changes in LBR binding states and oligomerization forms, resulting in chromatin rearrangement and altered gene expression (for a provisional model see Figure 3).

On the other hand, it is well-known that cholesterol levels have a critical impact on the organization and properties of membrane microdomains, known as lipid rafts [108]. Strikingly, it was suggested that cholesterol reaches the nucleus and participates in the assembly of nuclear lipid microdomains that may act as a platform for chromatin anchoring, thus regulating gene expression [109,110]. Given that LBR is actively involved in cholesterol biosynthesis, its sterol reductase activity may contribute to the production of cholesterol (and/or derivatives of cholesterol synthesis) at the microenvironment of NE, leading to the formation of INM lipid rafts, which may act as “niches” for the multimerized LBR molecules [21,22], but also for other chromatin tethers, such as the various lamin/integral INM–protein complexes [4,50,51]. These nuclear lipid rafts may, in addition, contribute directly to chromatin tethering, and it was recently suggested that cholesterol may influence chromatin condensation by directly binding to nucleosomes [111]. This may explain the unique role of LBR in cholesterol biosynthesis, which cannot be compensated for by the ER sterol reductase TM7SF2, even though both proteins catalyze the same reaction. LBR molecules residing in the ER may contribute to sustain the total cholesterol levels [21,86]. In line with this notion, Wassif et al. showed that LBR and TM7SF2 provide substantial enzymatic redundancy with respect to cholesterol synthesis [88].

The role of LBR in nuclear architecture may therefore rely on both its sterol reductase activity, which provides the necessary cholesterol and/or cholesterol derivatives to assemble nuclear lipid rafts, and its capacity to form functional oligomers, which are accommodated in these lipid microdomains and bind nuclear components essential to tether heterochromatin to the INM. This may explain why truncated forms of LBR that are degraded, and/or LBR mutants that result in complete loss of its sterol reductase activity, are associated with Greenberg dysplasia and are lethal in humans. For example, homozygosity for mutations R583Q (that leads to the production of full-length LBR lacking sterol reductase activity) and c.32delTGGT (that creates a premature stop codon) cause Greenberg dysplasia [87]. Conversely, an individual who was heterozygous for the missense mutation R583Q had the same amount of full-length LBR as a “normal”, non-mutant individual, and enough cholesterol (from the other copy of the gene) to support lobulation of neutrophil nuclei, and accordingly, did not show any evidence for the Pelger–Huët anomaly [87]. 

There are several structural and functional issues that need to be addressed in the upcoming future. Is LBR indeed associated with specific INM lipid “rafts”? What is the diffusional mobility in the INM of LBR mutants lacking sterol reductase activity? In other words, does the sterol reductase activity affect the oligomerization properties of LBR molecules? Are the sterol reductase-deficient LBR mutants organized in microdomains? What type, and how extensive, is the chromatin rearrangement that occurs in cells carrying these mutants? Does a crosstalk exist between the *N*- and *C*-terminal activities? How do post-translational modifications within the *N*-terminal nucleoplasmic domain (phosphorylation, O-GlcNAcylation, and others) affect the sterol reductase activity? Which are the signals that regulate the sterol reductase activity? Giannios et al. suggested that intra-molecular interactions exist among the loop segments that connect the transmembrane domains to each other [21]. If such interactions exist, how do they affect the sterol reductase activity and/or adjust the overall configuration of LBR molecules? Answering the above fundamental questions will help to unravel the molecular mechanisms underlying the complex crosstalk between the structure and function of the LBR complex, and furthermore, will shed light on how the expression/repression of particular genes is regulated at the nuclear periphery.
